# Serum MGP and ECP are commonly elevated in first-onset and recurrent ischemic stroke: a two-phase DIA proteomic study in a Chinese cold-region cohort

**DOI:** 10.3389/fneur.2026.1885191

**Published:** 2026-09-29

**Authors:** Ningning Wang, Zhengguang Guo, Ming Jin, Nan Wu, Zhi Zheng, Wei Sun, Zaihua Zhang, Changchun Qiu, Keyong Zhang

**Affiliations:** 1 Institute of Polygenic Disease, Qiqihar Medical University, Qiqihar, China; 2 Institute of Basic Medical Sciences, Chinese Academy of Medical Sciences and Peking Union Medical College, Beijing, China; 3 Mingzhu Hospital, Qiqihar, Heilongjiang, China

**Keywords:** biomarker panel, ECP, ischemic stroke, MGP, proteomics, recurrent stroke

## Abstract

**Background:**

Ischemic stroke (IS) has a high recurrence rate, yet effective biomarkers for distinguishing first-onset from recurrent IS remain limited. This study aimed to identify serum proteomic biomarkers commonly elevated in both first-onset and recurrent IS and to evaluate their ability to discriminate each disease state from healthy controls in a Chinese cold-region population.

**Methods:**

The study was conducted in two phases. In the discovery phase, 10 patients with first-onset IS, 10 with recurrent IS, and 10 healthy controls were analyzed using Data-Independent Acquisition (DIA) proteomics; the validation phase expanded the sample to 49 patients with first-onset IS, 30 with recurrent IS, and 50 healthy controls. Differentially expressed proteins were defined as fold change≥1.5 or ≤0.67. Multivariable Firth penalized logistic regression adjusted for age and sex constructed diagnostic models, with data randomly split into training and test sets (2:1) for receiver operating characteristic (ROC) analysis.

**Results:**

Two core proteins were identified: matrix Gla protein (MGP) and eosinophil cationic protein (ECP). Both were significantly elevated in the first-onset and recurrent IS groups compared with healthy controls (all *p* < 0.05). Two separate logistic models incorporating these proteins discriminated first-onset IS (test-set AUC 0.993) and recurrent IS (test-set AUC 0.959) from healthy controls, confirmed by repeated 10-fold cross-validation; the two proteins did not differ significantly between the patient groups. Enrichment analysis of their interaction partners revealed involvement in neutrophil extracellular trap formation, inflammatory responses, and immune regulation.

**Conclusion:**

Serum MGP and ECP were commonly elevated in both first-onset and recurrent IS, and two separate combinatorial models discriminated each disease state from healthy controls with high accuracy. This biomarker panel covers three core pathological processes-atherosclerosis and vascular calcification, chronic inflammation, and hypercoagulable state-providing candidate biomarkers that warrant further investigation for disease-state characterization and risk stratification, particularly in high-latitude cold-region populations.

## 1 Introduction

Cerebral infarction (ischemic stroke) is a common type of cerebrovascular disease. Every year, approximately 795,000 people suffer from strokes, of which 76.7% are first occurrences and 23.3% are recurrent cases (1). Among them, ischemic stroke accounts for a large proportion, reaching 62.4% (2). The same trend applies in China, with 3.94 million new strokes in 2019 and 2.19 million deaths (3). Cold exposure can increase the risk of cerebral infarction through pro-inflammatory and platelet-activating pathways (4). Although antiplatelet, anticoagulant, and comprehensive management of blood pressure, blood sugar, and blood lipids have to some extent reduced the recurrence rate, a survey conducted in China from 2015 to 2018 showed that the recurrence rate within 1 year after cerebral infarction still reached 12.5% (5), which has become a serious public health issue. More importantly, the first and recurrent ischemic strokes should not be regarded as two stages of the same disease process: the mortality, disability level, hospitalization time, and medical expenses of recurrent cases are all higher, and secondary prevention and close follow-up need to be strengthened; for the first cases, accurate risk stratification is required to prevent recurrence. Therefore, the clinical significance of the two is different: first-onset diagnosis focuses on acute identification, while recurrence prediction and monitoring involve secondary prevention and prognosis management. Molecular markers that can reliably distinguish these two states will directly serve these two purposes.

Currently, the distinction between first-onset and recurrent ischemic stroke is almost entirely dependent on the patient’s medical history and neuroimaging examinations. However, this approach has significant limitations: silent cerebral infarctions may not be detected, thereby leading to an underestimation of the recurrence risk; the medical history is often incomplete or unreliable, especially in elderly patients or during the acute phase; and imaging cannot reveal the underlying pathological and physiological state of the blood vessels and circulatory system, which determines the recurrence risk. Although there is increasing evidence that blood biomarkers can be used to track the long-term risk of recurrent stroke (6), no serum protein panel has been established to characterize the molecular features of first-onset and recurrent IS or to monitor recurrence risk, and it remains undefined whether the two disease states share common serum proteomic alterations. This is the specific clinical gap that this study aims to address.

The expression levels of proteins directly participate in the occurrence of diseases and respond to internal and external stimuli. Blood contains information about local and systemic events before and after stroke, which can be used to screen for biomarkers for stroke onset and recurrence. Therefore, studying the molecular mechanisms of diseases from the perspective of proteomics is of great significance. In recent years, with the continuous development of high-throughput proteomics technologies, significant progress has been made in the research on the pathogenesis, diagnosis, prevention, and treatment of cerebral infarction. Indicators such as retinol binding protein 4 (RBP4), matrix metalloproteinases (MMPs) (7), glutathione S-transferase Pi 1 (GSTP1) (8), etc., have been reported to be associated with the occurrence of ischemic cerebral infarction. Moreover, most of these reported markers—such as glial fibrillary acidic protein (GFAP) (9) and heart-type fatty acid-binding protein (FABP) (10)-mainly reflect acute parenchymal injury after stroke onset rather than the chronic vascular and immune processes that determine recurrence, limiting their value for disease-state characterization and recurrence monitoring. A particularly relevant study by Zhang et al. (11) investigated protein signatures associated with ischemic-stroke recurrence in 214 patients from the Third China National Stroke Registry. It used DIA-based discovery and targeted MRM validation and identified subtype-dependent signatures involving TAGLN2, ITGAM, MYL9, RSU1 and COL1A2 (11). However, most of the previous studies were aimed at detecting the occurrence of a single type of ischemic stroke, or merely focused on the subtypes, rather than reflecting the biological differences between the first occurrence and the recurrence events. Moreover, the reproducibility of these candidate markers in different analysis platforms and study populations is still inconsistent. Most importantly, there is currently no study comparing the serum protein profiles of patients with the first episode and recurrent ischemic stroke in the same cohort, nor has a specialized comparative proteomics analysis for the first episode and recurrent cerebral infarction been conducted in the Heilongjiang population of China—this is precisely the specific knowledge gap that this study aims to fill.

Emerging evidence suggests that vascular calcification is an active and regulated process closely related to plaque instability and thrombosis; first, matrix Gla protein (MGP) is a vitamin K-dependent inhibitor of arterial calcification, associated with carotid plaque calcification and poor prognosis in patients with cerebrovascular diseases (12), while functional MGP gene variations increase the susceptibility to ischemic stroke (13). Second, eosinophil-mediated inflammation has become an important factor in atherosclerotic thrombosis: eosinophil cationic protein (ECP) released during eosinophil activation is associated with the presence of carotid plaques and the incidence of stroke (14). Third, neutrophil extracellular trap (NET) formation (NETosis) promotes immune thrombosis: during NET formation, nucleosome histones (including variants of histone H1, such as H1.5) are released into the circulation, and elevated NET load has been observed in thrombi and circulation of patients with acute ischemic stroke (15, 16). The three processes of atherosclerosis, chronic inflammation, and hypercoagulable state are candidate pathophysiological axes that may differ between the first and recurrent ischemic stroke; however, whether circulating proteins reflecting these axes are commonly or differentially altered in first-onset and recurrent cerebral infarction remains unknown.

Therefore, this study employed the serum proteomics technology based on data-independent acquisition (DIA)-a technique that has demonstrated good reproducibility and quantitative accuracy in the discovery of circulating biomarkers (17)-to systematically analyze the serum proteome of patients from the cold regions of China (Heilongjiang Province, approximately at 47°–53° north latitude). Cold exposure in this region independently increases the risk of cerebral infarction through pro-inflammatory and platelet activation pathways. The study was based on a two-stage independent cohort design, using logistic regression combined with ROC analysis to construct a combined diagnostic model, aiming to identify serum proteins elevated in both first-onset and recurrent IS and to construct and validate separate diagnostic models distinguishing each disease state from healthy controls. This study may provide candidate biomarkers that warrant further investigation for recurrence risk stratification and mechanistic exploration, and represents the first comparative serum proteomic analysis of first-onset and recurrent cerebral infarction in this cold-region population.

## 2 Materials and methods

### 2.1 Study participants

This study was reviewed and approved by the Medical Ethics Committee of Qiqihar Medical University (Approval Number: [2016]06). All participants signed the informed consent form, and the research process strictly followed the principles of the Helsinki Declaration.

This study adopted a two-stage independent cohort design: the Discovery phase (2019-2020, *n* = 30) and the expanded validation phase (2021–2023, *n* = 129), collecting samples separately, with a 2–4-year interval between the two stages, but both coming from the same region (Qiqihar, approximately at 47°N). The two stages were completely consistent in terms of blood collection, coagulation, centrifugation, preservation, Filtration Assisted Sample Preparation enzymatic digestion, and quality control (QC) (QC *R*^2^ = 0.985/0.986; each sample underwent only one freeze–thaw process), and were all completed by the same institution and laboratory using the same standard operating procedures (SOP), Data-Independent Acquisition Proteomics (DIA) process, and Liquid Chromatography–Tandem Mass Spectrometry (LC–MS/MS) platform, thereby eliminating inter-laboratory differences. The data from the discovery stage and the validation stage were not combined and were analyzed independently to ensure that the validation stage truly played the role of independent verification.

The study subjects were Han people living in high-latitude areas ranging from 47°N to 53°N. Inclusion criteria were patients with ischemic stroke diagnosed by computed tomography (CT) or magnetic resonance imaging (MRI), aged 60 years or younger. Exclusion criteria included: (1) hemorrhagic stroke or transient ischemic attack; (2) previous history of stroke or other neurological diseases; (3) severe comorbidities (such as malignant tumors, renal failure, liver dysfunction); (4) use of anticoagulant or antiplatelet drugs before the stroke; (5) infection or inflammatory diseases within the past 1 month. These criteria were applied equally to first-time and recurrent patients, and remained consistent throughout the discovery and expanded validation phase. For recurrent patients, new ischemic foci must be located in different vascular regions or occur in new areas unrelated to the primary stroke; this definition does not include early neurological deterioration due to the progression of the primary stroke.

The control group consisted of healthy individuals with normal blood pressure, blood lipids, and blood glucose. Through voluntary and free comprehensive physical examinations, individuals matched in region, age, gender, and ethnicity to the ischemic stroke patients without blood-related ties were selected. The control group underwent the same test items as the case group, without performing imaging examinations.

During the Discovery phase, 10 patients with the first-onset of ischemic stroke (53.6 ± 4.03 years) and 10 patients with recurrent ischemic stroke (53.6 ± 3.95 years) were recruited from the Neurology Department of Qiqihar Mingzhu Hospital. Additionally, 10 healthy control subjects without a history of cerebral infarction (51.4 ± 4.62 years) were selected from the Physical Examination Department of the same hospital. Among the 30 participants in the discovery stage, half were male. After matching by age and gender, proteomic analysis was conducted. Subsequently, in the expanded validation phase, experiments were carried out on an expanded sample set, including 49 patients with the first onset, 30 recurrent patients, and 50 healthy controls, with an average age of 57.17 ± 9.24 years.

### 2.2 Protein extraction and digestion

Regarding sample collection and processing, the patients in the experimental group had their blood samples collected in the early morning of the day after admission, while the control group collected their samples during the initial physical examination. After blood collection, the samples were left to stand at room temperature for 30 min to coagulate, and then centrifuged at 3000 rpm at 4 °C for 15 min; the serum was aliquoted and stored at −80 °C for future use. For quality-control purposes, an additional 2 mL of serum was taken from each ischemic stroke sample and pooled as the quality control (QC) sample, which was regularly injected into the LC–MS/MS system to monitor repeatability.

The concentration of serum proteins was determined using the Bradford method, and a membrane-assisted sample preparation method (FASP) was employed for protein digestion: the serum samples were reduced at 95 °C with 20 mM DL-Dithiothreitol (DTT) for 10 min, then alkylated at room temperature in the dark with 50 mM Iodoacetamide (IAM) for 45 min; a solution containing 25 mM sodium bicarbonate was added and the samples were digested overnight at 37 °C.

### 2.3 Reagents and instruments

Ammonium bicarbonate, IAM, DTT, formic acid, trifluoracetic acid, and high-performance liquid chromatography (HPLC)-grade acetonitrile (ACN) were purchased from Sigma-Aldrich (St. Louis, MO, United States). Sequencing-grade trypsin was purchased from Promega (Madison, WI, United States).

An Orbitrap Exploris 480 Mass Spectrometer (Thermo Fisher Scientific, Bremen, Germany) coupled with an Ultimate 3,000 UHPLC (Thermo Fisher Scientific) system was used in the discovery phase.

### 2.4 Definition of differentially expressed proteins

Differentially expressed proteins (DEPs) were defined as upregulated when abundance was increased ≥1.5-fold and as downregulated when abundance was reduced to <0.67-fold, relative to the healthy group.

### 2.5 Protein–protein interaction (PPI) analysis

To identify potential interacting partners of the two core proteins (MGP and ECP), protein–protein interaction analysis was performed using the STRING database (https://string-db.org, version 12.0) with *Homo sapiens* as the species. A combined confidence score threshold of 0.4 was applied to retain high-confidence interactions. The 20 interacting partners were retrieved, yielding a network of 22 proteins (including the two query proteins). The resulting interaction data were then subjected to Gene Ontology (GO) and Kyoto Encyclopedia of Genes and Genomes (KEGG) pathway enrichment analyses using Metascape.

### 2.6 Bioinformatics analysis

For the GO analysis, Metascape^1^ was used for GO enrichment analysis. For Ingenuity Pathway Analysis (IPA), the SwissProt accession numbers were uploaded to the IPA software (Ingenuity Systems, Mountain View, CA, United States). The proteins were mapped to the disease and function categories, canonical pathways available in the Ingenuity knowledge base, ranked by *p*-value.

### 2.7 Statistical analysis

All statistical analyses were performed using R software (version 4.3.2). Continuous variables were presented as mean ± standard deviation (SD). Comparisons among the control, first-onset IS, and recurrent IS groups were performed using one-way analysis of variance (ANOVA), followed by post-hoc tests when appropriate. Binary logistic regression was used to construct diagnostic models for distinguishing first-onset and recurrent IS from healthy controls. The ROC curve was plotted and the area under the curve (AUC) was calculated to evaluate the model performance. The 95% confidence interval was obtained using the DeLong method. To assess the model stability and reduce the risk of overfitting, the caret package in R was used for 10-fold cross-validation for each model, repeated five times (total of 50 folds). In each cycle, the model was trained on 90% of the data and evaluated on the remaining 10% of the data. To avoid information leakage, all feature selection steps (missing value filtering, abundance filtering, and univariate ROC screening) were strictly completed within each sampling fold, ensuring that the information of the test fold does not affect feature selection or model fitting (18, 19). Hypertension, diabetes mellitus, SBP, and DBP were considered consequences or accompanying features of ischemic stroke (IS) rather than independent exposures, and were therefore not included as covariates. Accordingly, only age and sex were adjusted for in the subsequent analyses. To control confounding factors, a multivariate logistic regression model was additionally fitted, and the adjusted odds ratio (OR) and its 95% confidence interval (CI) were reported. The inter-group comparison of baseline features was conducted using ANOVA, Kruskal-Wallis, or *χ*^2^ tests, depending on the data distribution. A two-sided *p*-value less than 0.05 was considered statistically significant.

## 3 Results

### 3.1 Baseline demographic and clinical characteristics of the three groups

Table 1 presents the baseline demographic and clinical characteristics of the study participants. Compared with healthy controls, age, systolic blood pressure (SBP), and diastolic blood pressure (DBP) were significantly higher in both the first-onset and recurrent groups (all *p* < 0.001). Body mass index (BMI) was 22.84 (21.16–25.03), 23.66 (22.36–25.35), and 24.22 (22.38–25.17) kg/m^2^ in the three groups, respectively, with no significant differences among them. In the patient groups, hypertension was documented in 25 (50.0%) and 6 (18.2%) patients, and diabetes mellitus in 13 (26.0%) and 3 (9.1%) patients, respectively; these comorbidities were not applicable to the healthy controls. In the present study, hypertension, diabetes mellitus, SBP, and DBP were considered consequences or accompanying features of ischemic stroke (IS) rather than independent exposures, and were therefore not included as covariates. Accordingly, only age and sex were adjusted for in the subsequent analyses.

**Table 1:** Baseline demographic and clinical characteristics of the study participants.

Characteristic	Healthy controls (*n* = 50)	First-onset IS (*n* = 49)	Recurrent IS (*n* = 30)
Age, years	50.0 (46.2–54.0)	57.0 (54.0–59.8)***	67.5 (61.0–71.0)***
Male sex, *n* (%)	38 (76.0%)	39 (78.0%)	21 (65.6%)
SBP	115 (110–118)	140 (130–160)***	140 (130–160)***
DBP	77.0 (71.2–80.0)	90.0 (80.0–100.0)***	80.0 (80.0–90.0)***
BMI, kg/m^2^	22.84 (21.16–25.03)	23.66 (22.36–25.35)	24.22 (22.38–25.17)
Hypertension, *n* (%)	—	25 (50.0%)	6 (18.2%)
Diabetes mellitus, *n* (%)	—	13 (26.0%)	3 (9.1%)

Compared with the healthy control group, ***p < 0.001.

### 3.2 Differential proteomics analysis in the discovery phase

A total of 30 patients were included in the discovery phase, including 10 in the first-onset IS group, 10 in the recurrent IS group, and 10 in the healthy control group. All 30 serum samples were analyzed using the DIA method, and a total of 384 proteins were identified.

The technical differences in the analysis were evaluated by calculating the coefficient of variation (CV) of the protein abundance of 5 quality control replicate samples (*R*^2^ = 0.985, Figure 1A). The results showed that 296 proteins with a CV less than 0.5 were retained for the identification of differentially expressed proteins (Figure 1B).

**Figure 1:**
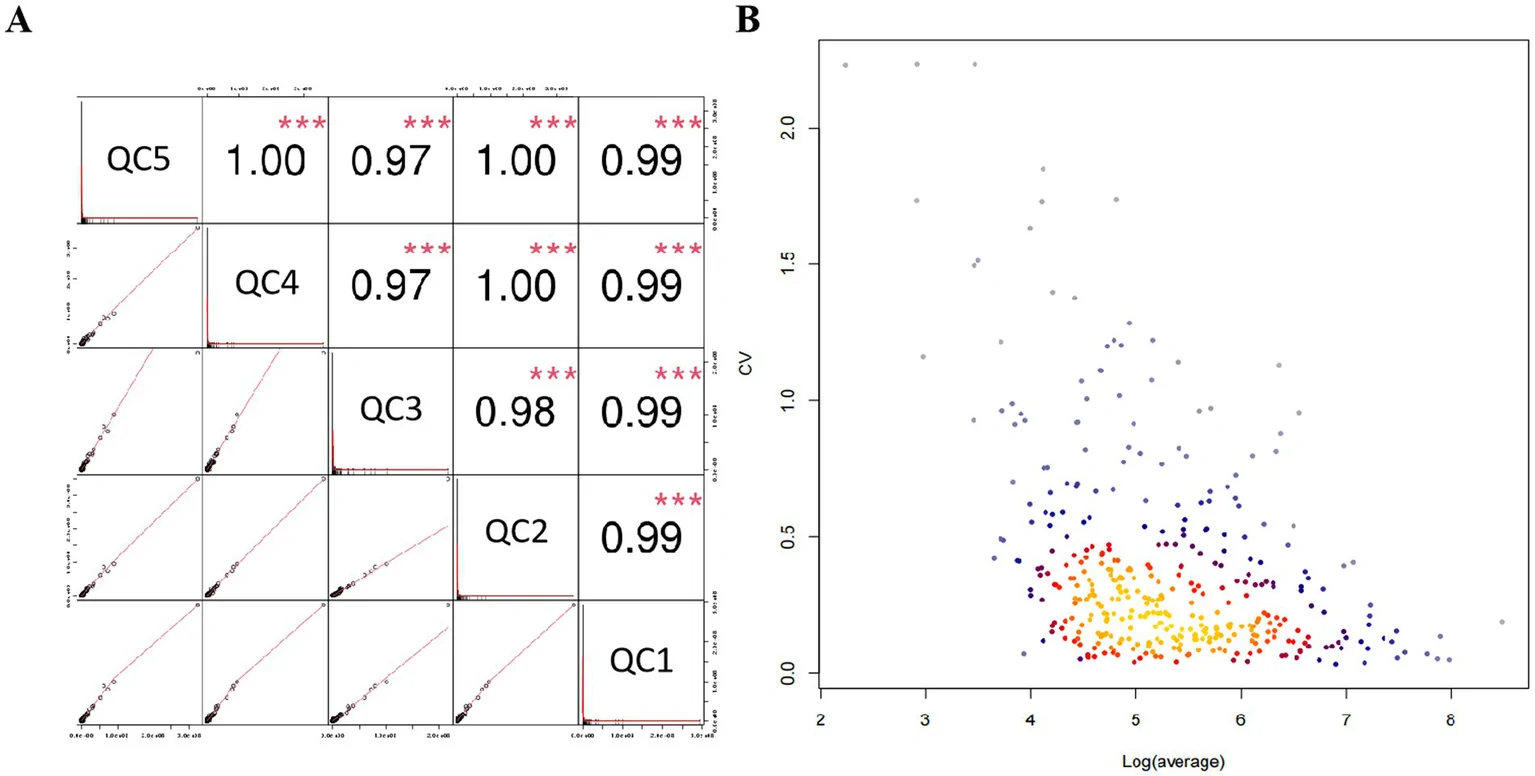
Reproducibility of the DIA analysis in the discovery phase. **(A)** Correlation analysis of pooled QC injections (*n* = 5 QC replicates). **(B)** Distribution of protein-wise coefficients of variation (CVs). CV, coefficient of variations; QC, quality control; DIA data-independent acquisition.

To explore the proteomic differences among these three groups, Partial Least Squares Discriminant Analysis (PLS-DA) was used. The score plots showed significant differences in proteomic characteristics among the three groups (Figure 2A). These results suggested that these differences in protein expression had classification and discrimination value. Figures 2C,D present volcano plots depicting differentially expressed proteins between the first-onset stroke group and controls and the recurrent stroke group versus controls, respectively.

**Figure 2:**
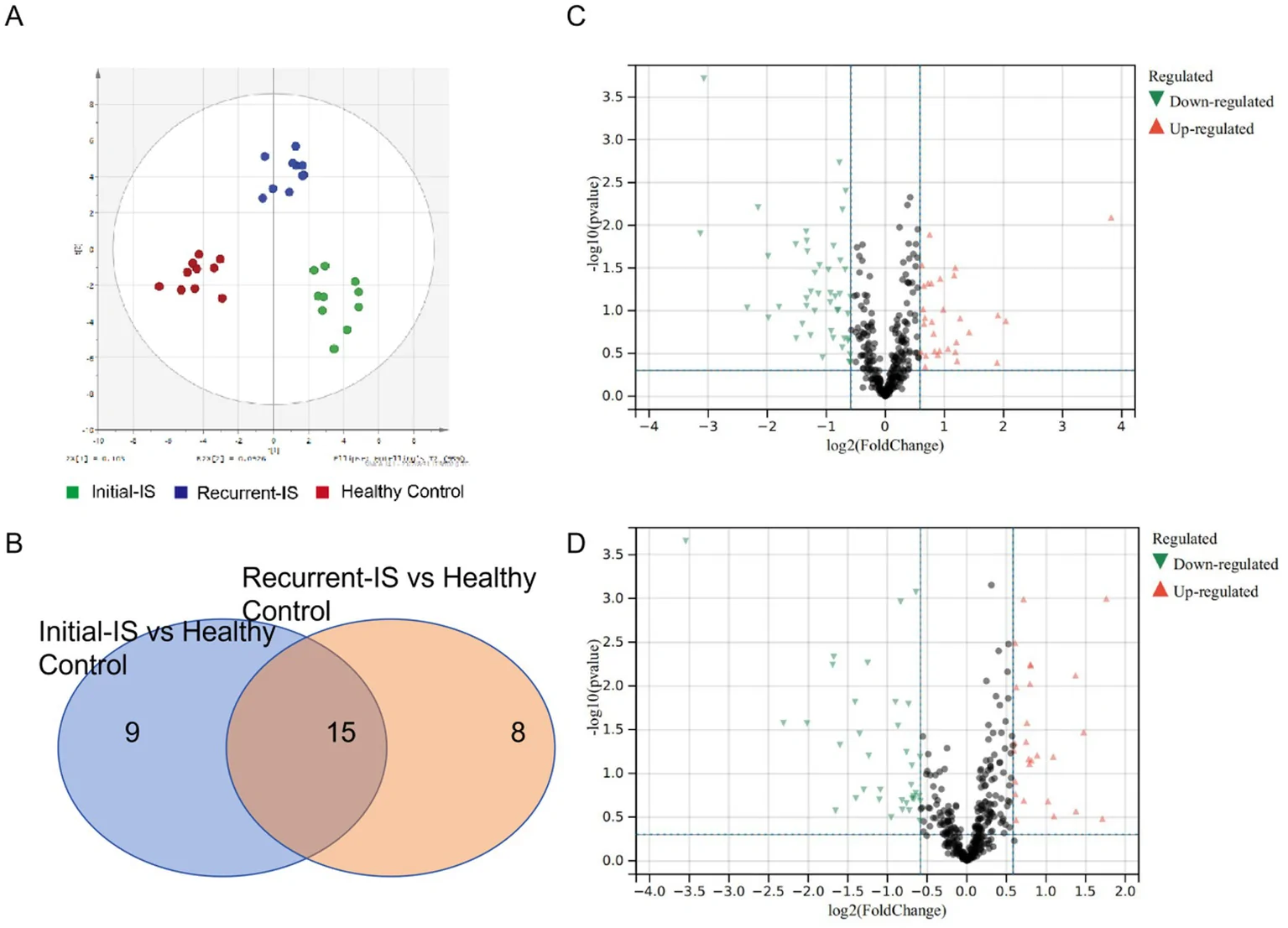
Proteomic profiling of the discovery phase (*n* = 10 first-onset IS, 10 recurrent IS, 10 healthy controls). **(A)** Score plot of the PLS-DA model among the first-onset IS, recurrent IS, and healthy control groups. **(B)** Venn diagram of the identified differentially expressed proteins. **(C,D)** Volcano plots of differentially expressed proteins in the first-onset IS and recurrent IS groups compared with the healthy control group; the *x*-axis represents log2 FC and the *y*-axis represents −log10 (*p*-value). DEPs were defined by FC ≥1.5 or ≤0.67 with BH-corrected *p* < 0.05.

To further clarify the differentially expressed proteins, in the expanded validation phase with an expanded sample size (49 cases in the first-onset cerebral infarction group, 30 cases in the recurrent cerebral infarction group, and 50 cases in the healthy control group), with a threshold of 1.5-Fold Change, the first-onset and recurrent cerebral infarction groups were compared with the healthy control group, respectively. In addition, t-tests were performed with Benjamini-Hochberg (BH) correction to increase the stringency of the differential protein screening. The results showed that 24 proteins were identified in the first-onset IS group and 23 proteins in the recurrent IS group. Among these proteins, 15 proteins were common in both the first-onset IS group and the recurrent IS group (Figure 2B).

### 3.3 Differential proteomics analysis in the expanded validation phase

At this stage, a total of 129 patients were included, including 49 patients in the first-onset IS group, 30 patients in the recurrent IS group, and 50 healthy controls. All serum samples were processed using the same experimental methods and screening mechanisms as in the discovery phase.

The technical differences in the analysis were evaluated by calculating the CV of the protein abundance of 4 quality control replicate samples (*R*^2^ = 0.986). The protein feature distribution among the three groups was analyzed by OPLS-DA, which could clearly distinguish them (Figures 3A, C). Meanwhile, after ANOVA and t-tests with BH correction, 128 proteins were identified in the first-onset IS group and 81 in the recurrent IS group. Among these proteins, 59 proteins were common in the first-onset type-IS group and the recurrent type-IS group (Figure 3D). The heatmap constructed based on 150 differentially expressed proteins related to IS showed the different protein profiles of the three groups of samples (Figure 3B).

**Figure 3:**
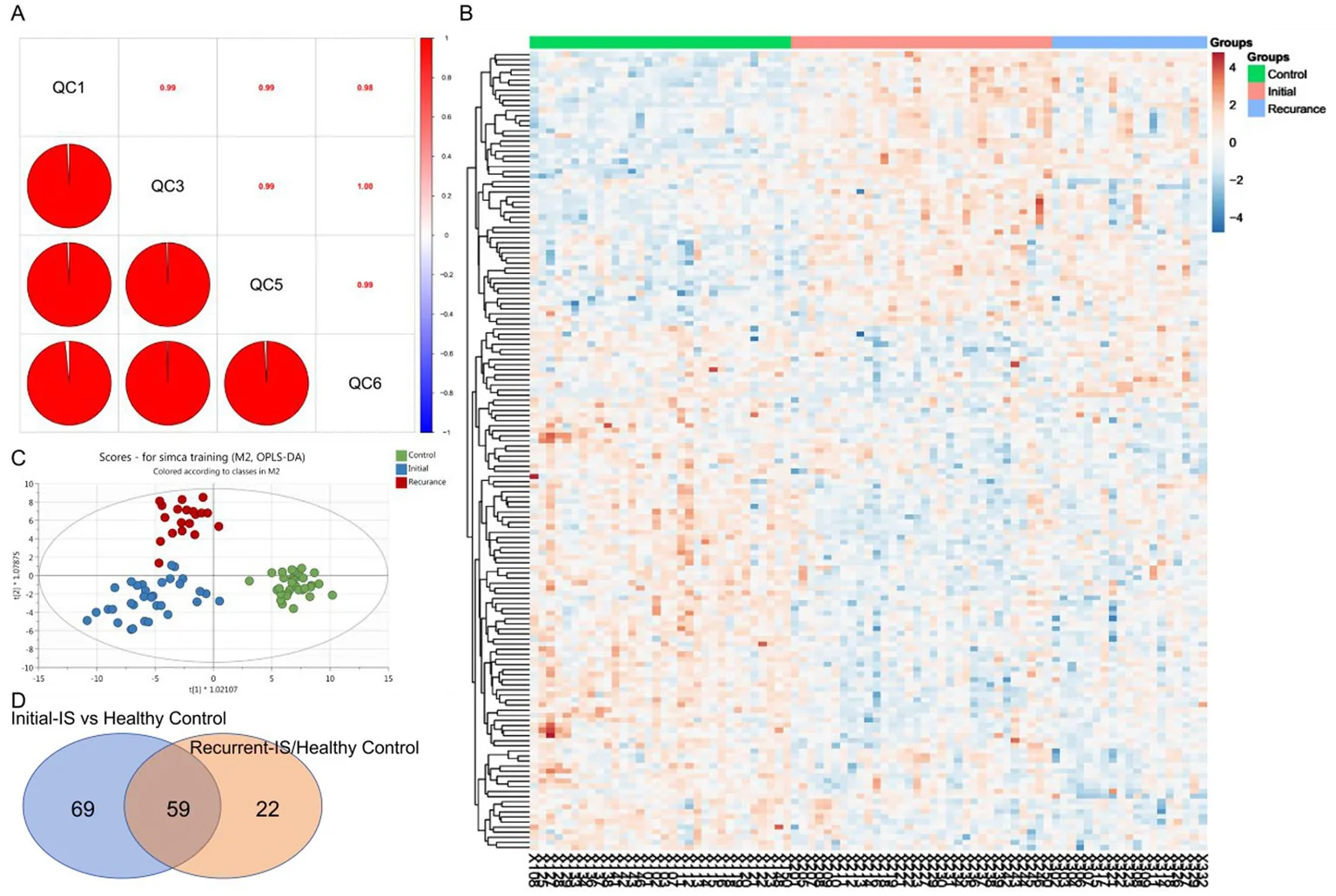
Differential proteomics analysis in the expanded validation phase (*n* = 129: First-onset IS *n* = 49, recurrent IS *n* = 30, healthy controls *n* = 50). **(A)** Score plot of the OPLS-DA model among the three groups. **(B)** Venn diagram of the identified differentially expressed proteins. **(C)** Heatmap of the 150 ischemic-stroke-related differentially expressed proteins across the three groups. **(D)** Volcano plots of differentially expressed proteins; the *x*-axis represents log2 FC and the *y*-axis represents −log10 (*p*-value). DEPs were defined by FC ≥1.5 or ≤0.67 with BH-corrected *p* < 0.05 (two-sample *t*-test with BH correction; one-way ANOVA for three-group screening).

### 3.4 Screening of protein biomarkers and model combination for first-onset ischemic stroke

To further screen the proteins that have an impact on both first-onset and recurrent cerebral infarction in the two independent phases, a Venn diagram was used to integrate and analyze the results of the differentially expressed proteins in the two experiments. The results showed that there were 12 proteins related to first-onset cerebral infarction in both phases (Figure 4A), and 11 proteins related to recurrent cerebral infarction in both phases (Figure 4B). Among the proteins related to first-onset cerebral infarction, six up-regulated proteins (Immunoglobulin kappa variable 1–39 (IGKV1-39), Antileukoproteinase (SLPI), MGP, Thioredoxin (THIO), ECP, histone H1.5) were obtained by screening for proteins with a missing value ratio of no more than 30% and an abundance ranking in the top 90%. To further screen for differentially expressed proteins, the validation-phase data were randomly divided into a training set and a test set at a ratio of 2:1, and ROC analysis was performed on the above six proteins. The results showed that the AUC of MGP, ECP and H1-5 remained consistent in both the training set and the test set, and were all ≥0.8 (Figures 4C–F). To adjust for potential demographic confounding, multivariable Firth penalized logistic regression models were constructed. After adjustment for age and sex, MGP (adjusted OR per SD 52.7, 95% CI 9.0–309.5) and ECP (adjusted OR per SD 5.2, 95% CI 2.4–11.5) each remained significantly associated with first-onset IS (both *p* < 0.001) in single-marker models.

**Figure 4:**
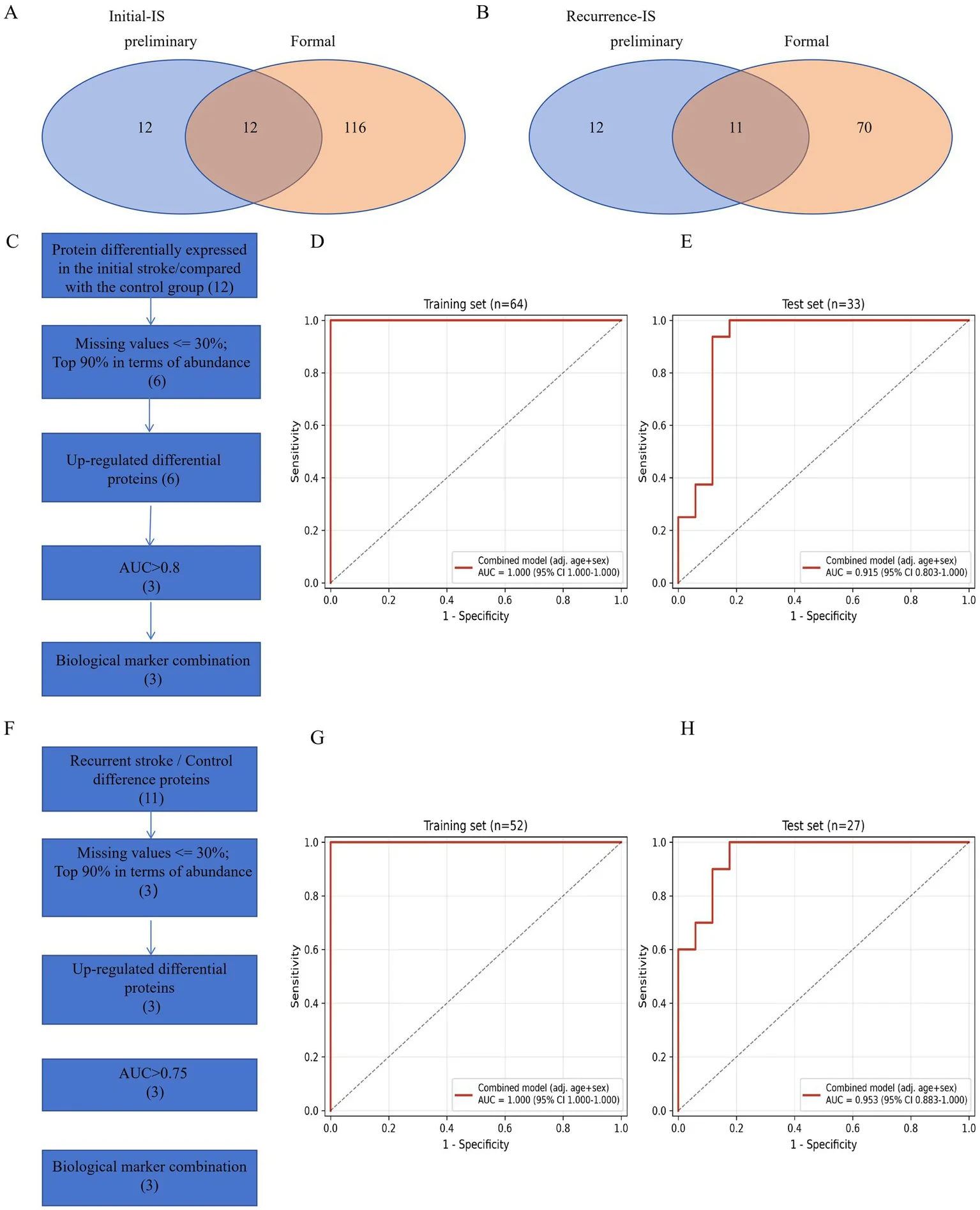
Integrated screening of core protein biomarkers across the discovery and expanded validation phases. **(A)** Venn diagram integration of the differentially expressed proteins from the two phases. **(B)** Screening of proteins related to first-onset IS and recurrent IS. **(C)** Biomarker-screening workflow for first-onset IS. **(D,E)** ROC analysis of the combined biomarker panel in the training and test sets for first-onset IS. **(F)** Biomarker-screening workflow for recurrent IS. **(G,H)** ROC analysis of the combined biomarker panel in the training and test sets for recurrent IS.

Although H1-5 was also significant in the single-marker model (adjusted OR per SD 9.1, 95% CI 3.4–24.5) (Figures 4F–H), it showed no independent association with first-onset IS in the combined model (adjusted OR per SD 2.5, 95% CI 0.8–7.9, *p* = 0.126), and its addition to the MGP + ECP model did not significantly improve discriminative performance (paired DeLong test, *p* = 0.327 for the full-sample fit; out-of-fold AUC 0.953 vs. 0.955). H1-5 was therefore excluded from the final model.

In the final combined model incorporating MGP, ECP, age and sex (logit(*P*) = −21.280 + 22.864 × MGP + 14.866 × ECP + 0.185 × age − 0.085 × female), both MGP (adjusted OR per SD 629.4, 95% CI 16.2–2.4 × 10^4^, *p* < 0.001) and ECP (adjusted OR per SD 15.8, 95% CI 2.7–93.1, *p* = 0.002) remained independent predictors. In a stratified 2:1 split (training *n* = 64, test *n* = 33), the final model achieved an AUC of 0.993 (95% CI 0.975–1.000) in the test set, with sensitivity 100.0% and specificity 94.1% at the Youden-optimal cutoff. Repeated stratified 10-fold cross-validation (5 repeats) confirmed model stability (mean fold AUC 0.985 ± 0.029; pooled out-of-fold AUC 0.953, 95% CI 0.902–1.000).

### 3.5 Screening of protein biomarkers and model combination for recurrent ischemic stroke

The same screening steps as those for first-onset IS were applied to recurrent IS. Among the 11 differentially expressed proteins, three proteins strongly associated with recurrent cerebral infarction were ultimately retained, which were consistent with the key differentially expressed proteins of first-onset IS, namely MGP, ECP, and H1-5 (Figure 4F). To adjust for potential demographic confounding, multivariable Firth penalized logistic regression models were constructed. After adjustment for age and sex, MGP remained significantly associated with recurrent ischemic stroke (adjusted OR per SD 96.6, 95% CI 1.7–5.4 × 10^3^, *p* = 0.026 in the final model), whereas ECP (adjusted OR per SD 2.7, 95% CI 0.7–9.8) and H1-5 (adjusted OR per SD 2.0, 95% CI 0.7–5.9) showed positive but non-significant associations, likely reflecting the limited number of recurrent cases (*n* = 30).

Furthermore, adding ECP or H1-5 to MGP did not improve discriminative performance (full-sample AUC 0.995 for MGP alone vs. 0.997 and 0.998 for the extended models, paired DeLong *p* = 0.281 and 0.277, respectively; out-of-fold AUC 0.982 vs. 0.980 and 0.980). ECP and H1-5 were therefore not retained in the final model, and the single-marker model was preferred to avoid overfitting given the small number of recurrent cases. Age was independently associated with recurrent IS (adjusted OR 1.38 per year, 95% CI 1.10–1.72). In a stratified 2:1 split (training *n* = 52, test *n* = 27), the final model achieved an AUC of 0.959 (95% CI 0.884–1.000) in the test set, with sensitivity 90.0% and specificity 94.1% at the Youden-optimal cutoff. Repeated stratified 10-fold cross-validation (5 repeats) confirmed model stability (mean fold AUC 0.993 ± 0.024; pooled out-of-fold AUC 0.982, 95% CI 0.958–1.000).

### 3.6 Expression differences of the two proteins between the first-onset and recurrent groups

In both the discovery and expanded validation phases, the expression levels of MGP and ECP were compared among the control group, the first-onset IS group (Figures 5A,B) and the recurrent cerebral infarction group (Figures 5C,D). The results showed that in both phases, the expression levels of these two protein biomarkers in the first-onset IS group and the recurrent cerebral infarction group were significantly higher than those in the control group (all *p* < 0.001). Moreover, there was no significant difference in the expression levels of these two proteins between the first-onset IS group and the recurrent cerebral infarction group.

**Figure 5:**
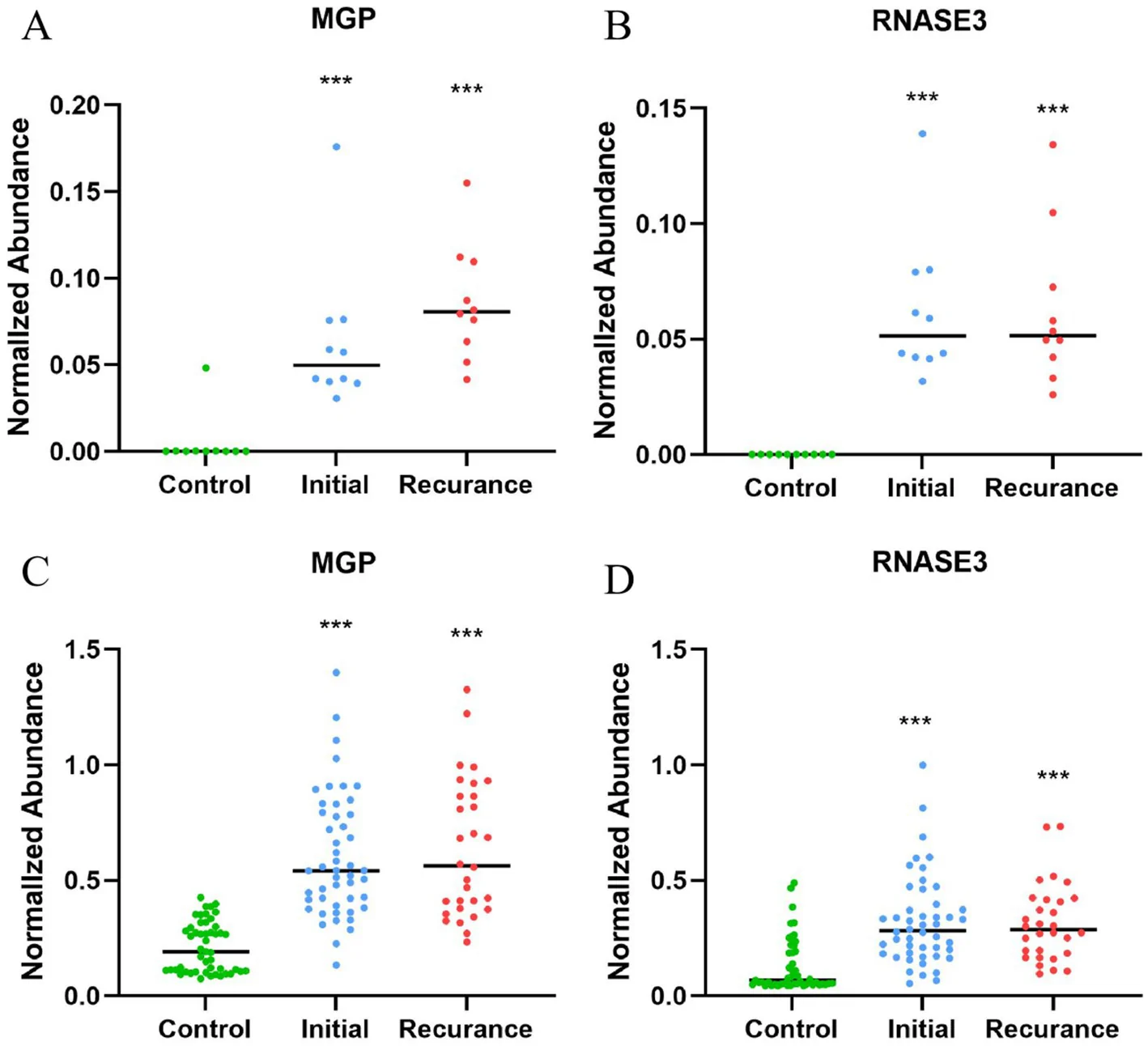
Serum levels of MGP and ECP among healthy controls, patients with first-onset IS, and patients with recurrent IS. **(A,B)** Discovery phase (*n* = 10/10/10). **(C,D)** Expanded validation phase (*n* = 49 first-onset IS, 30 recurrent IS, 50 healthy controls). Boxes indicate the median and interquartile range. The three groups are color-coded (blue, first-onset IS; red, recurrent IS; green, healthy controls), and median lines are drawn in black for visibility. Groups were compared by Kruskal-Wallis *H* test; exact *p*-values are shown.

### 3.7 Protein–protein interaction and pathway enrichment analysis

To explore the functional interactions of MGP and ECP, protein–protein interaction (PPI) analysis was performed using the STRING database. Separate queries using MGP and ECP as seed proteins yielded two high-confidence protein networks (Figures 6A,B). The MGP-centered network (Figure 6A) comprised 10 interacting partners, including PROS1, GGCX, VKORC1, BGLAP, ABCC6, BMP2, SPP1, ELN, AHSG and UCMA. The ECP-centered network (Figure 6B) comprised 10 interacting partners, including RNASE2, IL5, EPX, PRG2, ELANE, MPO, MS4A3, AZU1, CTSG and ANG. A total of 22 proteins (2 seed proteins and 20 interacting partners) were then subjected to enrichment analysis (Figure 6C). The top biological process (BP) terms included defense response to bacterium, biomineralization and regulation of biomineralization. Cellular component (CC) analysis showed enrichment in vesicle lumen, cytoplasmic vesicle lumen and secretory granule lumen. Molecular function (MF) enrichment highlighted sulfur compound binding, glycosaminoglycan binding and heparin binding. KEGG pathway analysis identified neutrophil extracellular trap formation, asthma and ubiquinone and other terpenoid-quinone biosynthesis as the top pathways (bubble size indicates gene count; color indicates adjusted *p*-value, with red reflecting higher adjusted *p* values).

**Figure 6:**
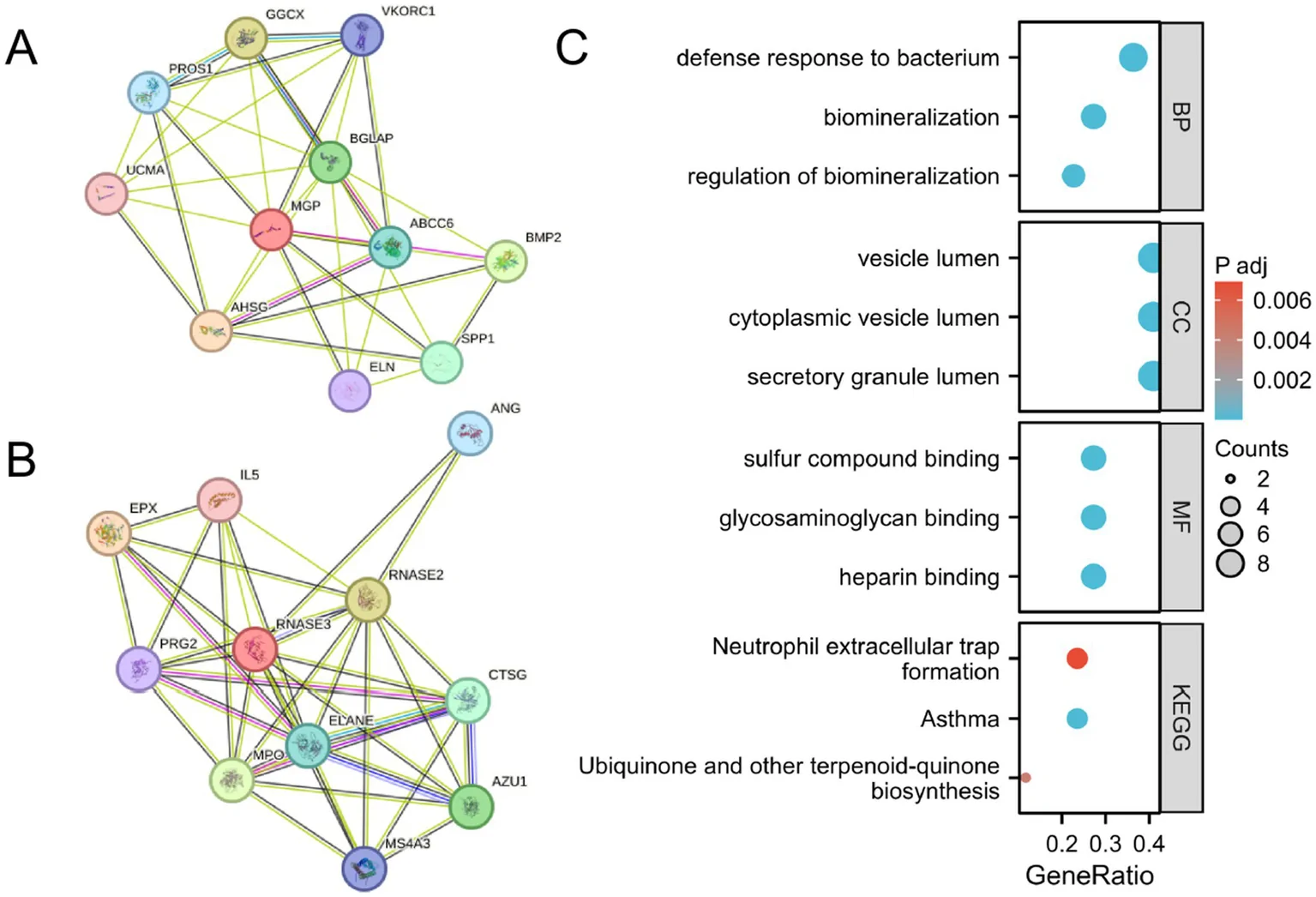
Protein–protein interaction networks and functional enrichment analysis of the identified proteins and their interactors. **(A,B)** PPI networks of MGP and ECP, respectively, retrieved from the STRING database (combined confidence score ≥0.4). **(C)** GO and KEGG pathway enrichment results for the 22-protein interaction network.

## 4 Discussion

This study, targeting the patients with first-onset and recurrent stroke in the cold regions of northern China and the healthy control group, adopted a two-stage DIA design to conduct parallel serum proteomics comparisons. It was found that in both stages, the two proteins MGP and ECP effectively discriminated first-onset ischemic stroke from healthy controls (test-set AUC 0.993) and recurrent ischemic stroke from healthy controls (test-set AUC 0.959), and their performance was stable in repeated 10-fold cross-validation. It is noteworthy that there were no significant differences in these two proteins between the first-time and recurrent groups. This indicates that these two disease states show a common increase in inflammation/thrombosis-related characteristics rather than a specific distinction between them.

At the biological level, the two candidates map onto two complementary pathological processes. MGP is a vitamin K-dependent inhibitor of arterial calcification expressed in vascular smooth muscle cells, chondrocytes, and endothelial cells (20), and impaired MGP function has been linked to vascular calcification, arterial stiffness, and cerebrovascular prognosis (21, 22); previous studies have shown that genetic variability in the MGP gene was associated with vascular recurrence in the Spanish population (23).

ECP is released during eosinophil activation and can mediate platelet aggregation (24), and eosinophil infiltration has been documented in atherosclerotic lesions (25, 26), a phenomenon analogous to eosinophilic airway inflammation (27). Notably, several interacting partners of ECP identified in our PPI analysis (e.g., ELANE, MPO, CTSG, PRG2 and AZU1) are neutrophil/eosinophil granule proteins, and the enrichment of neutrophil extracellular trap (NET) formation in the KEGG analysis is consistent with recent evidence of hyperacute NET formation in the cerebral circulation after ischemic stroke (28) and of the prognostic value of circulating NET markers (29). NET formation in turn promotes coagulation activation, linking the inflammatory signature captured by our panel to the hypercoagulable state characteristic of acute IS.

Together, these processes—atherosclerosis and vascular calcification, chronic inflammation, and hypercoagulability—provide a coherent working hypothesis for the joint elevation of the two proteins in both disease states, although direct mechanistic inference is beyond the scope of this cross-sectional study.

Finally, the cold-region setting of this cohort should be interpreted as a population-context descriptor rather than as evidence of cold-specific mechanisms. The cohort was recruited in Heilongjiang Province (47°N–53°N), where cold exposure is prevalent and has been associated with increased cerebral infarction risk through sympathetic activation, increased blood viscosity, platelet activation, coagulation–fibrinolysis imbalance, oxidative stress, and inflammatory responses (30–33), and where lifestyle factors such as high-salt, high-fat diets and reduced sunlight exposure may further aggravate metabolic and vascular damage (34, 35). Because no non-cold-region comparison group was included, this study cannot isolate cold-related effects; the cold-region context nevertheless defines a population with a high stroke burden in which specific serum biomarker data were previously scarce.

This study has several limitations. First, it is a single-center, discovery-stage proteomics investigation with a relatively modest sample size. Although the two-phase design (with temporally independent cohorts collected in 2019-2020 and 2021–2023) provides internal evidence of reproducibility, the findings have not yet been validated in an external cohort from a different institution or geographic region. Selection bias may therefore be present, and the generalizability of our results to other populations (particularly those outside high-latitude cold regions) remains uncertain. Second, the cross-sectional design precludes causal inference between the identified biomarkers and stroke recurrence. Third, the logistic regression models, although stable under 10-fold cross-validation, require verification using targeted proteomics approaches (e.g., parallel reaction monitoring or ELISA) in larger, multi-center, prospective cohorts before any clinical application. Furthermore, because the two patient groups were quantified in separately normalized DIA batches, a statistically valid direct first-onset-versus-recurrent model could not be constructed from the present data; pooling the two batch-normalized matrices would confound disease status with analytical batch. A dedicated direct comparison using batch-corrected raw data and targeted PRM quantification is planned. In addition, the original modeling workflow performed feature screening on the same data used for final model evaluation.

## 5 Conclusion

In this study, MGP and ECP were identified in serum using liquid chromatography–tandem mass spectrometry. These two proteins constitute an efficient biomarker panel whose value lies in covering three core pathological processes of ischemic stroke: atherosclerosis and vascular calcification, chronic inflammation, and a hypercoagulable state. Two logistic models derived from these two proteins discriminated each disease state (first-onset and recurrent IS) from healthy controls with high accuracy, whereas the serum levels of the two proteins did not differ significantly between the two disease states. This panel provides candidate biomarkers for disease-state characterization and risk stratification of ischemic stroke and warrants further validation in larger, multicenter cohorts.

## Data Availability

The raw data supporting the conclusions of this article will be made available by the authors, without undue reservation.

## Footnotes

1
http://metascape.org/gp/index.html
